# Systemic Sarcoidosis Presenting with Headache and Stroke-Like Episodes

**DOI:** 10.1155/2015/619867

**Published:** 2015-09-29

**Authors:** J. Campbell, R. Kee, D. Bhattacharya, P. Flynn, M. McCarron, A. Fulton

**Affiliations:** ^1^Department of Neurology, Royal Victoria Hospital, Belfast BT12 6BA, UK; ^2^Department of Neuroradiology, Royal Victoria Hospital, Belfast BT12 6BA, UK; ^3^Neurology Centre, Altnagelvin Area Hospital, Londonderry BT47 6SB, UK

## Abstract

Sarcoidosis is a multisystem granulomatous disorder. Neurological manifestations as a presenting symptom are relatively rare. A 26-year-old male presented with a five-week history of headache suggestive of raised intracranial pressure. He subsequently developed transient episodes of mild right-sided hemiparesis and numbness. Magnetic resonance imaging (MRI) of brain revealed widespread inflammatory white matter lesions, an ischaemic focus in the left corona radiata, and widespread microhaemorrhages consistent with a more diffuse vasculopathy. Serum angiotensin-converting enzyme (ACE) level was normal. Lumbar puncture revealed an elevated opening pressure (36 cmH_2_O) and inflammatory cerebrospinal fluid (CSF). Computerised tomography (CT) of chest, abdomen, and pelvis revealed widespread lymphadenopathy and biopsy of axillary lymph nodes revealed the presence of noncaseating granulomata in keeping with systemic sarcoidosis. The patient responded well to corticosteroids. This case highlights the importance of considering sarcoidosis to be a rare but potentially treatable cause of stroke in younger patients.

## 1. Introduction

Sarcoidosis is a multisystem granulomatous disorder characterised by noncaseating granulomata. The respiratory system is one of the most commonly involved sites and patients may present with cough and dyspnoea [1].

The nervous system is affected in approximately 5% of patients with sarcoidosis [2]. The neurological manifestations can be diverse but most commonly include cranial neuropathies (particularly involving the facial or optic nerves) [2, 3]. Other less frequent presentations include meningoencephalitis, hydrocephalus, intracranial mass lesions, psychiatric symptoms, spinal cord disease, peripheral nerve involvement, or even myopathy [4–7].

Despite pathological evidence of granulomatous involvement of cerebral blood vessels in neurosarcoidosis [8], stroke or transient ischaemic episodes are reported only very rarely. We report a case of an unusual presentation of systemic sarcoidosis with headache and stroke-like symptoms.

## 2. Case Report

A 26-year-old right-handed male was admitted to a district general hospital with a five-week history of gradual onset, dull, and global headache that was worse when supine and exacerbated by valsalva manoeuvres. There was associated nausea and frequent early morning vomiting. He also complained of prominent lethargy and approximately 5 kg of weight loss in the preceding four months.

He denied visual disturbance or other focal neurological symptoms at presentation. There was no history of febrile illness or recent travel. Soon after admission he experienced several transient episodes of sudden onset right upper limb weakness and numbness lasting up to 15 minutes without impairment of consciousness.

His past medical history was notable for an episode of right-sided uveitis six years prior to this presentation. He also had a history of well-controlled childhood epilepsy.

Approximately eighteen months prior to his current admission he was discovered to have renal impairment (urea 14.8 mmol/L and creatinine 226 umol/L) after a routine check-up revealed hypertension. He underwent renal biopsy that was consistent with interstitial nephritis and which was attributed to sodium valproate that he had been taking for treatment of epilepsy. The sodium valproate was switched to levetiracetam and he was started on prednisolone and lisinopril. Renal function subsequently stabilised, blood pressure normalised, and the prednisolone was gradually weaned over the following 18 months and had been stopped several weeks prior to this admission.

Medications on this admission were levetiracetam 2500 mg daily, omeprazole 20 mg daily, and lisinopril 2.5 mg daily.

## 3. Examination

On examination chest was clear on auscultation. There was no hepatosplenomegaly. On neurological examination tone, power, and coordination were normal. Reflexes were symmetrically brisk; however both plantar responses were flexor. Visual acuity was 6/6 in both eyes. On fundoscopy there was blurring of the optic disc margins bilaterally as well as absent spontaneous venous pulsations in both eyes. Blood pressure was 150/107. Systemic examination revealed the presence of diffuse, nontender cervical and axillary lymphadenopathy. There were no visible skin lesions. The remainder of the examination was normal.

Initial investigations included a mild normocytic anaemia (Hb 11.6 g/dL). Serum white cell count, erythrocyte sedimentation rate, C-reactive protein, liver function tests, and serum electrolytes (including calcium) were normal. Blood urea was 7.1 mmol/L; creatinine was 282 umol/L.

Other laboratory tests including tests for human immunodeficiency virus, VDRL, double stranded DNA, anti-nuclear antibodies, anti-neutrophil cytoplasmic antibodies, serum angiotensin-converting enzyme, antibodies to extractable nuclear antigens, anti-Ro, anti-La, anti-Sm, anti-RNP, anti-Scl, and anti-Jo1 as well as tuberculosis quantiferon and mantoux testing were normal or negative. Serology for herpes simplex virus, cytomegalovirus, Epstein-Barr virus, and varicella virus was negative. Chest radiograph was normal on admission.

CT brain on admission was normal. Cerebrospinal fluid analysis revealed an opening pressure of 36 cmH_2_O. CSF protein was 0.74 g/L and glucose was 2.9 mmol/L (no paired serum was sent for analysis). There were 9 lymphocytes/*μ*L and 72 erythrocytes/*μ*L. CSF was sterile. Oligoclonal bands were not detected.

**Figure 1:**
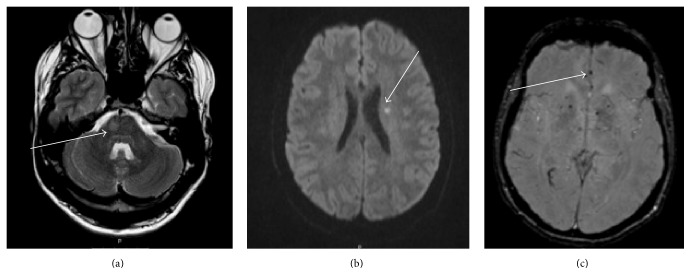
MRI scan of brain. MRI of brain. Axial T2-weighted image showing T2 hyperintense lesion within the right pons (arrow) (a). Axial DWI (B1000) demonstrates restricted diffusion within the left centrum semiovale (arrow) (b). Gradient recalled ECHO T2 susceptibility weighted imaging showing diffuse hypointense lesions suggestive of microhaemorrhages (arrow) (c).

MRI brain showed multiple small T2-weighted hyperintense lesions predominantly within the subcortical white matter throughout both cerebral hemispheres and also within the right pons. Microhaemorrhages were also observed on susceptibility weighted imaging (SWI) and an area of restricted diffusion was observed in the left centrum semiovale on diffusion weighted MRI consistent with a small acute infarct (Figure 1).

Repeat MRI imaging upon transfer to a tertiary neurology unit revealed further linear areas of T2 hyperintensity with postcontrast T1 enhancement extending from the periventricular regions into the centrum semiovale bilaterally.

**Figure 2:**
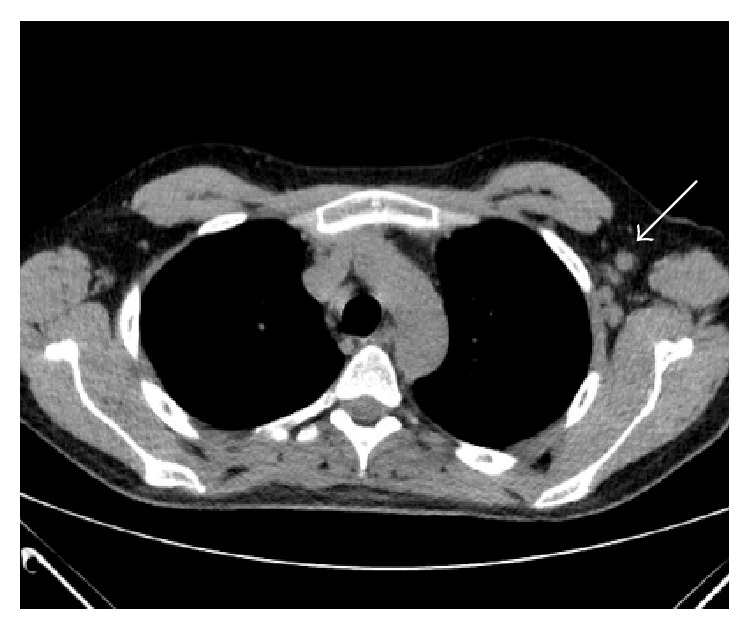
CT scan of thorax. CT of chest showing enlarged axillary nodes (arrow).

A differential diagnosis of cerebral vasculitis, a neoplastic process, or granulomatous disease was considered. CT of chest, abdomen, and pelvis revealed multiple lymph nodes in both axillae measuring up to 14 mm, as well as mediastinal and left para-aortic lymphadenopathy (Figure 2).

**Figure 3:**
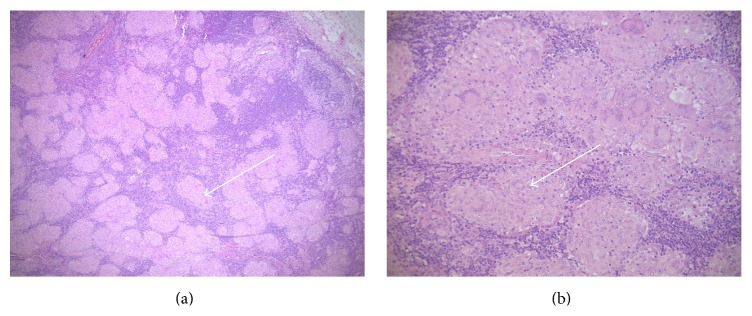
Axillary lymph node biopsy showing noncaseating granulomata (arrow) at ×50 magnification (a) and noncaseating granulomata (arrow) at ×200 magnification (b).

The patient underwent excision of one of the axillary lymph nodes, which revealed the presence of noncaseating granulomata in keeping with sarcoidosis (Figure 3). The original kidney biopsy was subsequently reviewed but did not contain evidence of granulomata.

## 4. Treatment and Outcome

The patient was treated with one gram of intravenous methylprednisolone for three days followed by 60 mg daily of oral prednisolone with rapid clinical improvement. Renal function improved to baseline following commencement of steroids. Repeat lumbar puncture one month following introduction of steroids revealed opening pressure of 19 cmH_2_O, protein 0.29 g/L, glucose 4.6 mmol, 2 lymphocytes/*μ*L, and 72 erythrocytes/*μ*L. Oligoclonal bands remained negative. At 12-month follow-up, the patient was well with no relapse of symptoms. He was maintained on 5 mg of prednisolone daily.

## 5. Discussion

Sarcoidosis is a systemic granulomatous disorder most commonly presenting between the ages of 20 and 40 years and is particularly prevalent among African Americans and Northern Europeans [9, 10]. The diagnosis of sarcoidosis most often relies on identifying systemic manifestations of the condition and obtaining histological demonstration of noncaseating granulomata on biopsy of affected tissue. Histologically, a central collection of macrophages is seen to exist with an oligoclonal T-cell population situated peripherally [11].

About 30–60% of patients with sarcoidosis develop granulomatous uveitis at some point in the course of the disease [12] but this can precede the systemic manifestations by up to eleven years [13]. Renal involvement may appear as interstitial nephritis and long-term treatment is often required to prevent progression to end stage renal failure [14].

Neurosarcoidosis is known to affect a minority of patients with sarcoidosis. The neurological features of sarcoidosis can be diverse and where neurological symptoms are the sole manifestation of the condition it can be particularly challenging to make a definitive diagnosis [15]. To further the investigation of suspected neurosarcoidosis, investigation with CSF analysis and measurement of CSF ACE levels are often undertaken. Both have limited sensitivity and specificity in the diagnosis of neurosarcoidosis [16]. Although tissue diagnosis remains the gold standard, it is invasive and not always feasible. MRI therefore remains an important investigation. The most common MRI findings in neurosarcoidosis include cranial nerve involvement, enhancing and nonenhancing parenchymal lesions, dural thickening, and leptomeningeal enhancement. Occasionally intracranial mass lesions are seen [17].

Our case is of interest for several reasons. The interval between the initial manifestations of sarcoidosis (presumed to be anterior uveitis) and subsequent symptoms was significant (over six years). The episode of acute onset right-sided weakness was shown to be related to an area of diffusion restriction on MRI. Focal ischaemic change in conjunction with MRI evidence of more diffuse microhaemorrhages was felt consistent with a vasculopathy. The neurovascular complications of neurosarcoidosis are extremely rare.

Although granulomatous involvement of cerebral blood vessels is described in neurosarcoidosis [8], stroke or transient ischaemic episodes are only rarely reported and such case reports largely precede the routine use of MRI [18–20]. Only isolated case reports exist of ischaemic stroke in the context of neurosarcoidosis confirmed on diffusion weighted MRI [21, 22]. Haemorrhagic manifestations of neurosarcoidosis are also reported but are a rare presentation [23–26]. Vascular complications of this nature are felt to result from a small-vessel vasculopathy, usually due to the destruction of elastic lumen by inflammatory cells and subsequent luminal occlusion. Acute necrotizing vasculopathy has also been observed [27].

The optimum treatment of neurosarcoidosis is unclear. Corticosteroids are generally considered the first-line treatment; however high doses may be required and relapse can occur during drug taper [28]. A number of agents including methotrexate, chloroquine, azathioprine, mycophenolate mofetil, cyclophosphamide, and infliximab can be used as second-line or steroid sparing agents [29–32]. Due to the rarity of neurosarcoidosis, large scale randomized controlled trial evidence is lacking.

## 6. Conclusion

We report a case of biopsy proven systemic sarcoidosis presenting with neurological features of raised intracranial pressure and transient neurological deficits, which were felt to be of vascular origin. Serial MRI over a short period showed significant change both revealing an area of restriction diffusion indicative of ischaemia and showing evidence of cerebral microhaemorrhages on susceptibility weighted imaging sequences as a result of small-vessel granulomatous vasculopathy.

In retrospect, the past medical history of anterior uveitis and interstitial nephritis is felt to be in keeping with systemic sarcoidosis and serves to highlight the considerable variation that can be seen in the natural history of this condition.

This case highlights that neurosarcoidosis should be considered a potentially treatable cause of otherwise unexplained vasculopathy or stroke, particularly in the context of other atypical neurological features or salient past medical history.
